# Considerations about the in situ derivatization and fractionation of EFA and NEFA in biological and food samples

**DOI:** 10.1016/j.mex.2015.11.006

**Published:** 2015-11-23

**Authors:** Lígia L. Pimentel, Ana L. Fontes, Ana M. Gomes, Luis M. Rodríguez-Alcalá

**Affiliations:** CBQF – Centro de Biotecnologia e Química Fina – Escola Superior de Biotecnologia, Universidade Católica Portuguesa/Porto, Rua Arquiteto Lobão Vital, Apartado 2511, 4202-401 Porto, Portugal

**Keywords:** EFA and NEFA in situ derivatization and fractionation, EFA, NEFA, Direct derivatization, Fatty acid fractionation, Biological samples, Foodstuffs

## Abstract

Despite their important role in tissues, fluids and foods, the analysis of non-esterified fatty acids (NEFA) as methyl esters (NEFAME) is performed using expensive, cumbersome and time-consuming procedures that needs of isolation, fractionation and derivatization steps. However, Yi et al. [1] proposed a promising in situ, single-step procedure to analyze esterified fatty acids (EFA) and NEFA from a same sample on the basis that acylglycerols and free fatty acids can be derivatized using specific reactions. However, according to the data presented in this research work, some modifications need to be performed to increase the reliability of the method:

• Increment of the transesterification performance by adding hexane to the reaction mixture, decreasing the time for the derivatization of acylglycerols from 10 min to 3–4 min and stopping the reaction with sulfuric acid.
• Avoid cross-contamination of the NEFAME extract by adding 500 μL of water after collection of EFA methyl esters (EFAME).
• Samples are spiked with three internal standards: a triacylglycerol (to calculate the concentration of EFA), a free fatty acid (to calculate NEFA) and a FAME (to control isolation of FAME and cross-contamination).

## Graphical abstract

In situ derivatization to methyl esters (FAME) and fractionation of EFA and NEFA.

**Figure:**
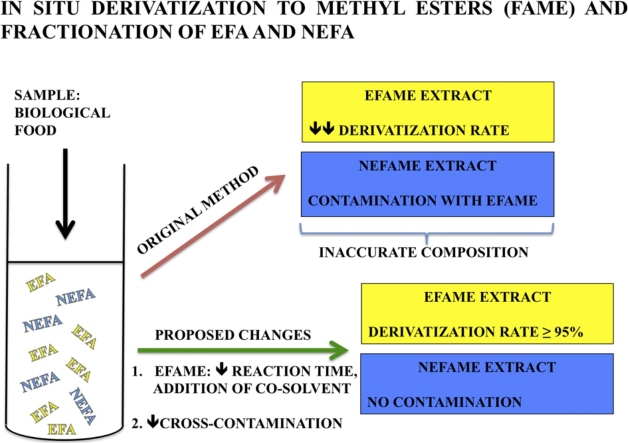

## Method details

All the modifications described in this work were carried out in order to increase the transesterification and esterification performance of fatty acids as well as to prevent cross contamination of the NEFAME extract with FAME from EFA when assaying the method described by Yi et al. [1].

## Chemicals

Hexane, dimethylformamide (DMF), methyl tert-butyl ether (MTBE) and methanol (MeOH) were HPLC grade and sulphuric acid was analytical grade (VWR Scientific, Carnaxide, Portugal). Supelco 37 FAME mix, methyl tricosanoate (99%; FAME-C23), methyl undecanoate (99%; FAME-C11), nonadecanoic acid (99%; FFA-C19), tung oil, sodium methoxide (MetNa; 95%) and potassium hydroxide (KOH) were obtained from Sigma (Sigma-Aldrich, St. Louis, MO, USA); GLC-Nestlé36 FAME mix, tritridecanoin (99%) and heptadecanoic acid (99%) were from Nu-Chek Prep, Inc. (Elysian, MN, USA). Undecanoic acid (99%; FAME-C11; ALFA AESAR, Karlsruhe, Germany) was obtained from VWR (Carnaxide, Portugal) while butterfat CRM-164 (EU Commission; Brussels, Belgium) was from Fedelco, Inc. (Madrid, Spain). Seronorm Lipid (Sero, Billingstad, Norway) was purchased to Bioportugal (Porto, Portugal). Earthoil (Bury St. Edmunds, Suffolk, UK) kindly donated the pomegranate oil (PMO). All the experiments were performed using 14 mL borosilicate glass tubes (16 mm × 125 mm) with acid/heat resistant cap (VWR international, Carnaxide, Portugal).

## Instrumentation

In Tests 1–3, EFAME and NEFAME were analyzed in a gas chromatrograph HP6890A (Hewlett-Packard, Avondale, PA, USA), equipped with a flame-ionization detector (GLC-FID) and a BPX70 capillary column (50 m × 0.32 mm × 0.25 μm; SGE Europe Ltd, Courtaboeuf, France). Analysis conditions were as follows: injector (split 10:1; injection volume 1 μL) and detector temperatures were 250 °C and 270 °C, respectively; carrier gas was Hydrogen (11 psi) and the oven temperature programme started at 60 °C (hold 2 min), raised 10 °C/min to 135 °C (hold 2 min), then 10 °C/min to 165 °C (hold 2 min) and finally 10 °C/min to 230 °C (hold 7 min).

For the analysis of EFAME and NEFAME in plasma and PMO samples (Test 4), due to the presence of *trans* fatty acids and conjugated linolenic acid isomers (CLnA), a 120 m × 0.25 mm × 0.25 μm BPX70 column (SGE Europe Ltd, Courtaboeuf, France) was installed in the HP6890 gas chromatograph. Conditions were, split 10:1, injection volume 1 μL, injector temperature 250 °C, detector (FID) temperature 290 °C, carrier pressure (Hydrogen) 30 psi while the oven programme was as follows: 70 °C hold 1 min, 7 °C/min to 170 °C (hold 41 min), 5 °C/min to 230° (hold 17 min).

Supelco 37, tung oil and CRM-164 were used for identification of fatty acids. GLC-Nestlé36 was assayed for calculation of response factors. All the preparations and analysis described in this research work were performed in triplicate.

## Statistics

In a first instance, data were examined for the presence of outliers and an exploratory analysis of data was performed to test normal distribution and homogeneity of variance (Levene's test). Then, in Tests 1, 2 and 4 comparison of data was accomplished through *t*-Student procedure. Comparison of data in Test 3 was performed using the ANOVA procedure with Bonferroni's or Tamhane's as post hoc according to homogeneity of variance. Analyses were conducted with the aid of the SPSS Statistics software v22.0 for Mac (IBM, Armonk, NY, USA). Level of significance was fixed at *p* < 0.05.

## Assays to improve the method

The mains drawback in the GC analysis of EFA and NEFA in biological and food samples is the need to include isolation and fractionation steps while its preparation or the utilization of hazardous reagents as diazomethane during the esterification of NEFA [2]. However, Yi et al. [1] reported in 2007 a direct derivatization/fractionation procedure avoiding such cumbersome and time-consuming steps on the basis that acylglycerols can be transesterified using mild conditions together with an alkali-catalyst while NEFA can be transformed into FAME using an acid catalyst [3]. Due to these a priori advantages over other techniques, the method was assayed in order to utilize it in further studies.

## Test 1: Assaying the transesterification and esterification ratios

During the adaptation of this method in our lab, a first experience was conducted using 100 μL of tritridecanoine (TG-C13; 1.3 mg/mL) and heptadecanoic acid (FFA-C17; 1.4 mg/mL) placed into the reaction tube and evaporated to dryness with a stream of nitrogen. A volume of 1 mL of the EFAME and NEFAME extracts was used for analysis and spiked with 100 μL of methyl tricosanoate (FAME-C23; 1.3 mg/mL). The original method used KOH in MeOH as base-catalyst but according to previous studies focused in the development of single-step derivatization methods [4], the utilization of MetNa at the same concentration than KOH in the original method as an alternative reagent and DMF as protectant added during the esterification reaction were assayed.

**Fig. 1:**
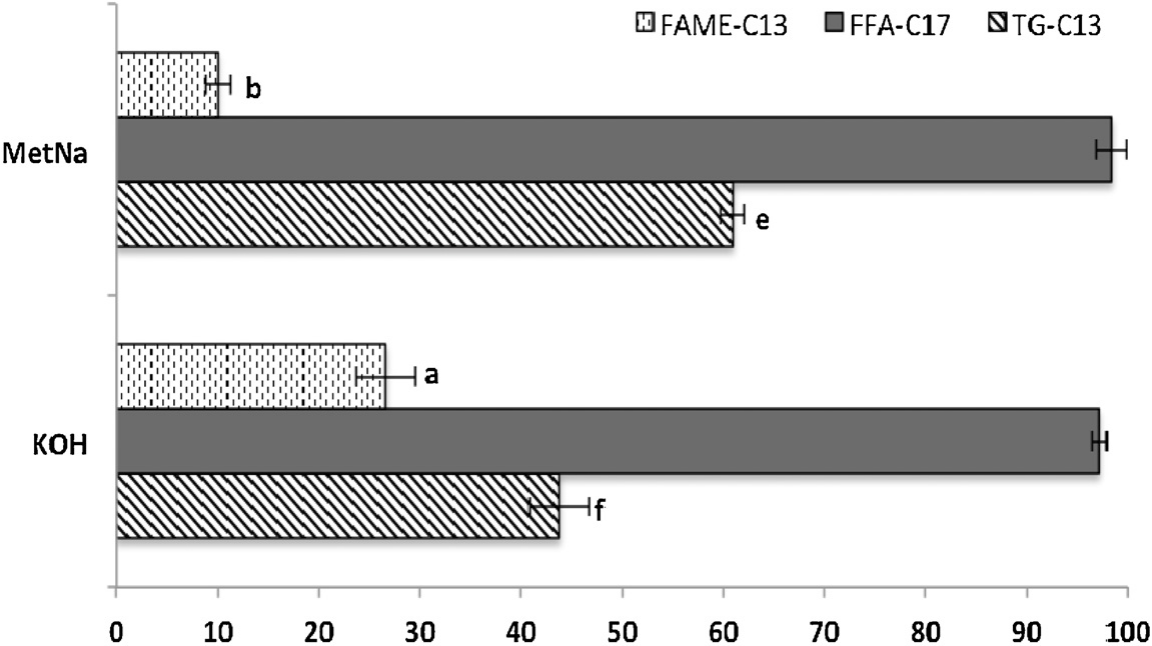
Transesterification (TG-C13) and esterification performance (FFA-C17) as well as cross-contamination of the NEFAME extract (FAME-C13).

The obtained results (Fig. 1) showed that transesterification of TG-C13 was incomplete: a 44% using KOH and 61% using MetNa (*p* < 0.05). Furthermore, in the NEFA extract it was detected the presence of FAME-C13 when using both reagents (27% in samples with KOH and 10% with MetNa; *p* < 0.05). The high esterification ratio for FFA-C17 implies that although this compound can dissolve in hexane, it has a better solubility in MeOH. Otherwise, when isolating the EFAME with hexane, would result in a high lost of FFA-C17.

Interestingly, the EFAME were collected with 4 mL of hexane and the recovered volume was recorded using graduated glass tubes. Thus, it was found that this extract has a final volume of 3.5 mL. This may point outs that 500 μL of hexane remained dissolved in the MeOH being the source of contamination in the NEFAME extract. Although specific reactions were used to derivatize EFA and NEFA, the final product is the same (FAME); therefore any contamination of the NEFAME extract with EFAME is not acceptable as the obtained composition would not be reliable. In the original method, samples were added with a standard mix of FFA-C17 and FAME-C17. This place some problems as an internal standard must have the same chemical characteristics of the compound to be quantified (e.g. a triacylglycerol for quantitation of acylglycerols, a free fatty acid for NEFA), otherwise, as our data show, the derivatization performance would not be considered during calculations. Furthermore, if a FAME is added, it should not be the same final product of any of the other standards as this will also affect the calculations or will not allow to detect cross-contaminations. At this point, the method needs some modification to increase the reliability of the data.

Other official methods intended for the analysis of EFA in dairy products proposed reaction times of 5 min [5] while some authors reported that times above 3–4 min may lead to the hydrolysis of the FAME, released as free fatty acids [6], [7]. These authors also concluded that the addition of an apolar solvent into the reaction mixture (e.g. hexane) increased the FAME/acylglycerols ratio.

## Test 2: Modifications to increase the effectiveness of FAME from acylglycerols

**Fig. 2:**
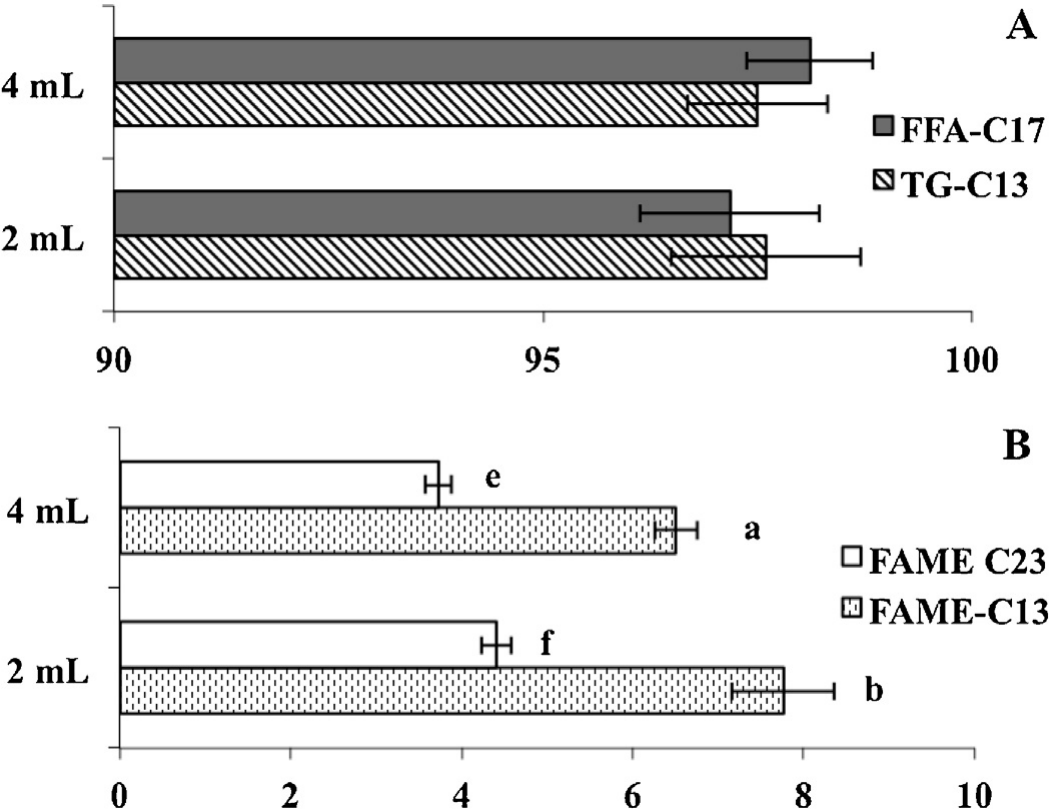
Effect of the modifications of the original method assayed in Test 2, in the transesterification using KOH as base-catalyst and esterification performance (A) as well as cross-contamination of the NEFAME extract (B).

The following experience (Fig. 2) was intended to increase the derivatization performance of the TG-C13 through decreasing the total reaction time to 3–4 min. For such purpose a mixture of TG-C13 (1.3 mg/mL), heptadecanoic acid (FFA-C17; 1.4 mg/mL) and FAME-C23 (1.3 mg/mL) was assayed and the final EFAME and NEFAME extracts were added with 100 μL of FAME-C11 (1.3 mg/mL). For a better control of the transesterification of acylglycerols, the reaction was stopped by adding 230 μL H_2_SO_4_ (3 M) in MeOH. The effect of the presence of hexane (2 mL vs. 4 mL) during the formation of EFAME was also studied. Furthermore, Castro-Gómez et al. [4] reported that for the derivatization of free fatty acids, 30 min at 30 °C were enough for their complete esterification. Therefore, such conditions were also tested using a dry-block giving a minimum temperature of 40 °C.

**Fig. 3:**
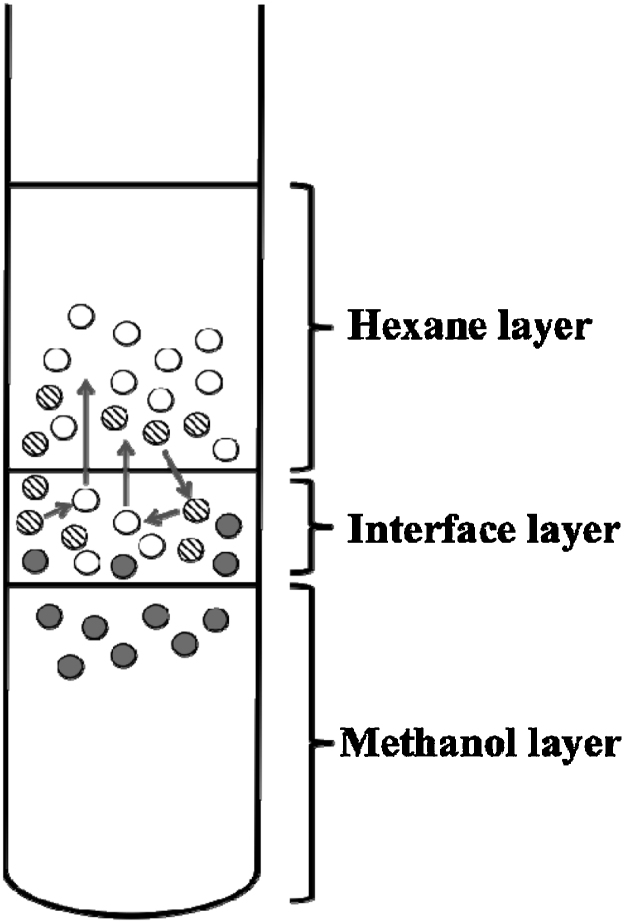
Distribution of EFA, NEFA and FAME during the transesterification reaction.

As it was not observed differences among the utilization of KOH or MetNa only data from KOH experiments are reported. According to the obtained results (Fig. 2A), the proposed modifications increased the transesterification performance (TG-C13) while it was not found significant differences associated to the addition of 2 or 4 mL in this parameter. The FAME-C23 was added to the reaction mixture in order to understand the behaviour of these kinds of compounds during the derivatization of EFA and the effectiveness of the isolation of the final products. It seems that methyl esters rapidly moved into the hexane layer. Araujo et al. [8], working in a method for the analysis of fatty acids from various matrices, proposed that the conversion of TG into FAME occurred in an *effective methylation area* (EMA), placed in the limit between the hexane and MeOH layers. However, on the basis of the results of this work, the EMA seems to be an interface layer where part of the hexane (dissolving some of the acylglycerols) and MeOH (dissolving the alkali and NEFA) mix (Fig. 3). It is also possible that compounds from the upper and lower layers can move to the interface. Thus, if some TG have to migrate from the hexane to the EMA, transform into FAME and them being transferred to the hexane layer again, it may explain that the concentrations of FAME-C13 in the NEFAME extracts were higher than those of the FAME-C23.

On the other hand, the assayed modifications lowered the amounts of FAME-C13 found in the NEFAME extract when compared with the results of the Test 1. Moreover, the higher the volume of hexane in the reaction mixture during step 1 the lower the contamination in terms of FAME-C13 and FAME-C23 in that extract (*p* < 0.05). The esterification performance for FFA-C17 was similar to those in the Test 1 in accordance with Castro-Gómez et al. [4].

In the following assays, transesterification was carried out in the presence of 4 mL and hexane while the conditions for the esterification were 30 min at 40 °C.

## Test 3. Effect of variation of solvent polarity in the reaction mixture

**Fig. 4:**
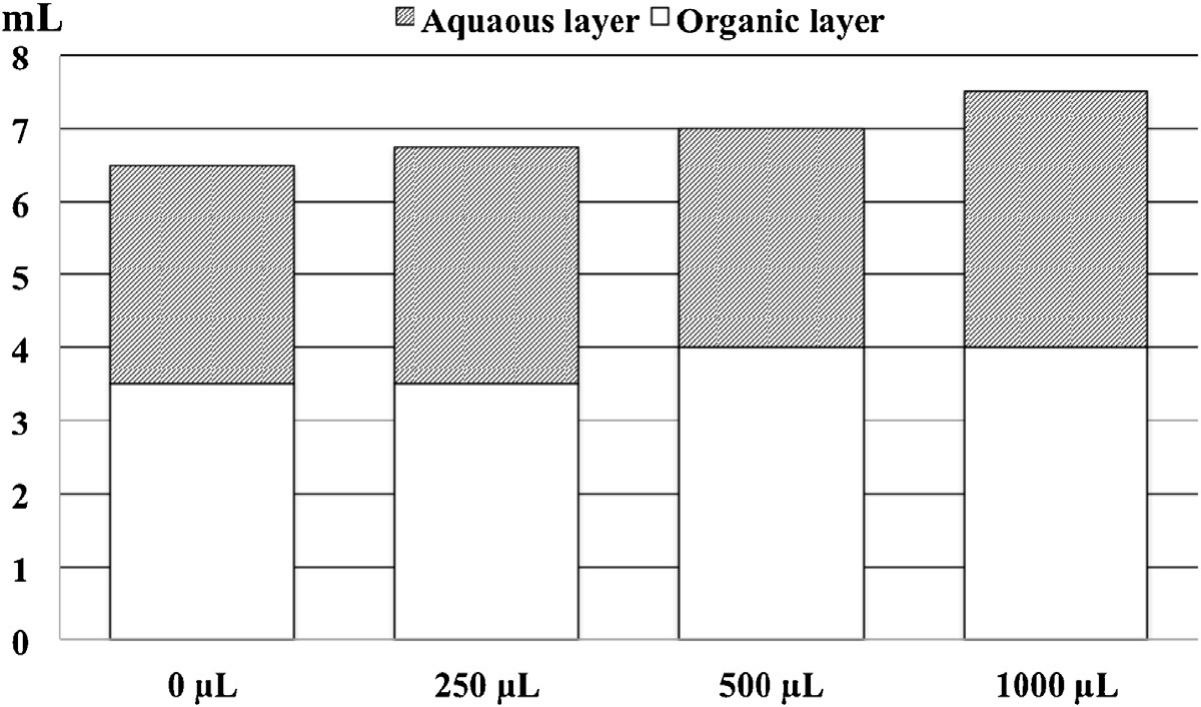
Effect of addition of water after collection of the EFAME extract in the total volume recovered.

The information from Tests 1 and 2 showed that the separation of the upper from the lower layer was not complete and that improvement of the derivatization ratio did not avoid the presence of FAME-C13 in the NEFAME extract. Therefore, the remaining volume of hexane in the methanol layer is the source of such contamination. In order to solve this drawback, the method was assayed (without sample) including a new step after collection of the 4 mL of hexane (EFAME extract): addition of 250, 500 or 1000 μL of water to increase the polarity of the methanol (Fig. 4). Thus, only by using 500–1000 μL was possible with a complete recovery of the 4 mL of hexane; therefore 500 μL water was assayed in further experiments. It was observed that this recovered volume of 500 μL of hexane had some traces of MeOH; thus it was discarded in further experiments.

**Fig. 5:**
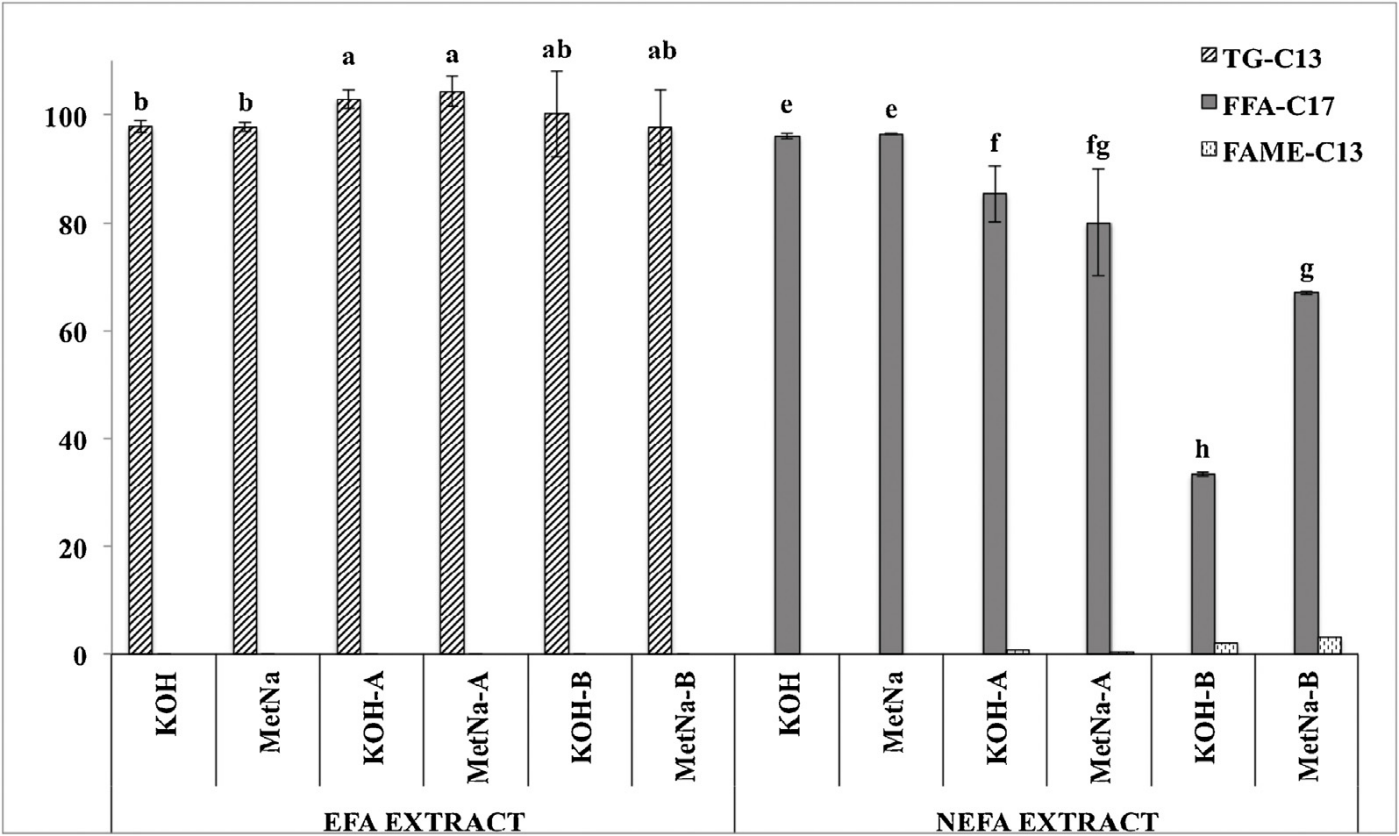
Derivatization performance (TG-C13 and FFA-C17) and contamination of the NEFAME extract (FAME-C13) resulting from Test 3.

Then the method was assayed with the same standard mix used in Test 2 (Fig. 5). Some authors reported the utilization of a mixture of hexane:MTBE (3:1) for the analysis of fatty acids in milkfat [7]. As polarity seems to play an important role in the derivatization performance and hexane recovery, the method was also tested using 4 mL of hexane:MTBE 3:1 (KOH-A; MetNa-A) and 1:3 (KOH-B; MetNa-B).

As in previous assays accomplished in this research work, it was not found differences from the utilization of KOH or MetNa in the production of EFAME. Although the hexane:MTBE 3:1 yielded transesterification ratios higher (*p* < 0.05) than when using only hexane, it resulted into a negative impact in the production of NEFAME (*p* < 0.05). Moreover, this latter parameter decreased with the amount of MTBE (*p* < 0.05). This can be explained as MTBE increased the solubility of NEFA in the hexane:MTBE mixture.

The addition of water, after isolation of the EFAME, resulted in the absence of contamination when this extract was composed only by hexane. However, the presence of MTBE resulted in 1% (3:1) and 3% (1:3) of FAME-C13 in the NEFAME extract (*p* < 0.05).

## Test 4. Assaying the final method with plasma and pomegranate oil samples

At this point, the method including all the modifications from Tests 1–3 (i.e. addition of 4 mL of hexane during transesterification and 3–4 min. of reaction time; addition of 500 μL of water for a full recovery of the EFAME extract; esterification conditions of 30 min at 40 °C) was assayed for the in situ preparation of EFAME and NEFAME using 500 μL of animal plasma and 5 mg of pomegranate oil (Table 1, Table 2). The procedure was also tested in order to know if the utilization of KOH or MetNa has any impact in the fatty acid composition. Thus, samples were spiked with 100 μL of TG-C13 and 100 μL of FFA-C19 (due to the presence of C17 in the pomegranate samples) both 1.3 mg/mL in hexane. EFAME and NEFAME extracts were added with 100 μL FAME-C11 (1.3 mg/mL), evaporated to dryness with a gentle stream of nitrogen and resuspended to 100 μL and 50 μL respectively.

The derivatization performance for TG-C13 and FFA-C19 was above the 95% without statistical differences for the utilization of KOH or MetNa as base-catalyst (data not shown). Furthermore, FAME-C13 was not detected in the NEFAME extracts in none of the assayed samples.

According to the obtained data, both KOH and MetNa resulted in similar EFA compositions (*p* < 0.05) as well as did not affected to the NEFA profile.

## Summary: Final proposed method

According to the results from Tests 1–4, the recommended modifications assure the reliability of the EFA and NEFA composition when comparing with the original method and these are consisting in:

1. Addition of 4 mL of hexane to the transesterification mixture.
2. Derivatization time for acyglycerols of 3–4 min, stopping the reaction with 230 μL H_2_SO_4_ (3 M) in MeOH.
3. Addition of 500 μL of water after collection of the EFAME extract.
4. Esterification conditions for NEFAME, 30 min at 40 °C.

Thus, summarizing the method:

1. Add internal standards into a glass tube with heat resistant cap: 100 μL of a TG (1.3 mg/mL) and 100 μL of a FFA (1.3 mg/mL) according to the composition of the sample.
2. Place the sample (5–10 mg (oil)/250 μL (e.g. plasma/milk)) + 2 mL MeOH + 4 mL hexane.
3. Vortex 30 s. Warm mixture at 40 °C in a dry-block.
4. Add 0.5 mL MetNa (2.5 M): reaction starts with the first drop and must not be longer than 3–4 m (Vortex frequently).
5. Stops reaction with 230 μL H_2_SO_4_ (3 M).
6. Cool into ice; separation of organic layers: 1250 g, 5 min, 25 °C.
7. Pipetting of 3–3.5 mL hexane to 15 mL Falcon; evaporate, resuspend to a proper concentration and put in a vial containing 100 μL of a FAME standard (1.3 mg/mL).
8. Eliminate the remaining hexane. Add 250–500 μL of water and eliminate. It is recommended not to pool as it may content traces of MeOH.
9. Wash the reaction medium with 2 mL of hexane. Vortex 30 sg; 1250 g, 5 min, 25 °C and discard.
10. Add 1.25 mL DMF + 1.25 mL 3 M H_2_SO_4_; 40–50 °C, 30 min (Vortex frequently).
11. Cool in ice; addition of 2–4 mL hexane to isolate NEFAME; Vortex 30 s; 1250 g, 5 min, 25 °C.
12. Recover a proper amount of the upper layer, evaporate to dryness with a gentle stream of nitrogen if need, redissolve using an appropriate volume and place into a vial containing 100 μL of a selected FAME standard (1.3 mg/mL).

## Figures and Tables

**Table 1:** EFA and NEFA composition in the assayed animal-based control serum.

Fatty acid	EFA				Fatty acid	NEFA			
	KOH (*n* = 3)		MetNa (*n* = 3)			KOH (*n* = 3)		MetNa (*n* = 3)	
	Mean	SD	Mean	SD		Mean	SD	Mean	SD
C14	4.00	0.10	3.92	0.23	C15ai DMA	0.73	0.07	0.76	0.11
C15ai	0.38	0.04	0.41	0.07	C15i DMA	0.41	0.08	0.41	0.11
C14:1 c9	0.50	<0.01	0.46	0.02	C14	3.86	0.42	3.91	0.58
C15i	0.74	0.05	0.65	0.09	C14 DMA	0.80	0.08	0.81	0.14
C15	1.55	0.14	1.44	0.13	C15	0.35	0.02	0.38	0.08
C16i	0.97	0.10	0.82	0.14	C16 DMA	4.69	0.67	4.80	0.74
C16	93.49	6.31	90.40	6.90	C16i	0.16	0.01	0.14	0.03
C16:1 c7	1.62	0.04	1.54	0.06	C15:1	0.48	0.05	0.50	0.08
C16:1 c9	5.31	0.09	5.17	0.30	C16	10.46	1.12	9.77	1.40
C17i	0.93	0.10	0.91	0.12	C16:1 c7	0.19	0.02	0.18	0.03
C17ai	1.55	0.16	1.53	0.14	C16:1 c9	0.81	0.07	0.73	0.08
C16 Phy	29.52	3.29	28.34	3.37	C17i	0.25	0.02	0.22	0.03
C16:2 c9t12	2.00	0.22	1.91	0.24	C17ai	0.34	0.01	0.33	0.04
C17	3.06	0.32	2.95	0.32	C16 Phy	0.58	0.06	0.51	0.04
C17:1 c9	0.45	0.01	0.39	0.10	C16:2 c9t12	0.12	0.02	0.10	0.03
C17:1 c10	0.59	0.05	0.52	0.08	C17	0.39	0.03	0.38	0.04
C18i	0.99	0.07	0.88	0.11	C18 DMA	0.89	0.15	0.86	0.10
C18ai	0.58	0.01	0.37	0.11	C17:1 c9	0.05	0.04	0.03	0.05
C18	89.86	9.12	86.78	8.94	C17:1 c10	0.11	0.04	0.12	0.03
C18:1 t9	0.59	0.18	0.40	0.01	C18i	0.22	0.05	0.21	0.07
C18:1 t10	0.29	0.03	0.23	0.01	C18ai	0.29	0.11	0.29	0.11
C18:1 t11	2.75	0.19	2.61	0.26	C18	9.84	0.86	9.11	0.89
C18:1 t12	2.13	0.12	1.97	0.21	C18:1 t9	0.41	0.10	0.42	0.08
C18:1 c9	117.31	5.10	113.07	6.69	C18:1 t10	0.09	0.02	0.09	0.02
C18:1 t15	0.49	0.03	0.47	0.05	C18:1 t11	0.52	0.09	0.51	0.09
C18:1 c11	4.74	0.18	4.62	0.30	C18:1 t12	0.25	0.04	0.25	0.05
C18:1 c12	1.13	0.08	1.09	0.13	C18:1 c9	10.92	1.86	10.13	1.69
C18:1 c13	0.64	0.02	0.61	0.10	C18:1 t15	0.14	0.03	0.16	0.02
C18:2 t9t12	0.72	0.11	0.61	0.04	C18:1 c11	0.59	0.09	0.56	0.06
C18:1 t16	0.41	0.12	0.36	0.13	C18:1 c12	0.13	0.01	0.15	0.02
C18:2 c9c12	88.74	7.95	85.96	7.98	C18:1 c13	0.12	0.02	0.17	0.02
C18:3 n6	1.25	0.11	1.19	0.15	C18:1 t16	0.11	0.01	0.16	0.02
C18:3 n3	11.92	1.30	11.70	1.38	C18:2 t9t12	0.29	0.06	0.21	0.02
C20	0.21	0.06	0.15	0.01	C18:2 c9c12	5.93	0.81	5.27	0.44
C18:2 c9t11	1.05	0.05	1.09	0.13	C18:3 n6	0.12	0.01	0.11	0.01
C20:1 c9	0.94	0.12	0.91	0.04	C18:3 n3	1.43	0.17	1.36	0.13
C21	1.29	0.33	1.26	0.09	C18:2 c9t11	0.32	0.14	0.32	0.10
C20:2 c11c14	0.94	0.17	0.96	0.10	C20:1 c9	0.50	0.11	0.41	0.06
C20:3 n3	10.74	1.30	10.45	1.27	C20:3 n3	0.56	0.09	0.46	0.05
C20:4 n6	13.64	1.62	13.33	1.55	C20:4 n6	0.97	0.17	0.81	0.08
C22:1 c7	5.63	0.90	5.79	0.93	C22:1 c7	0.50	0.08	0.35	0.08
C20:5 n3	5.06	0.61	4.87	0.57	C20:5 n3	0.59	0.06	0.59	0.09
C24	0.31	0.04	0.30	0.03	C24	0.58	0.23	0.50	0.07
C22:5 n6	1.40	0.13	1.36	0.15	C22:5 n6	0.20	0.03	0.21	0.01
C22:5 n3	5.76	0.74	5.51	0.66	C22:5 n3	0.46	0.09	0.37	0.04
C22:6 n3	4.29	0.48	4.16	0.50	C22:6 n3	0.31	0.03	0.26	0.05

mg/dL	522.48	41.63	504.40	45.41	mg/dL	62.05	8.04	58.37	7.94

ai: ante iso; i: iso; c/t: cis/trans double bond; Phy: phytamic acid; n6: ω6 fatty acid; n3: ω3 fatty acid; DMA: dimethylacetal.

**Table 2:** EFA and NEFA composition of the assayed pomegranate oil.

Fatty acid	EFA			
	KOH (*n* = 3)		MetNa (*n* = 3)	
	Mean	SD	Mean	SD
C16	30.91	1.39	29.52	<0.01
C16:1 c9	0.60	0.03	0.61	0.04
C17	0.72	0.02	0.67	0.03
C18	25.28	0.82	24.66	0.20
C18:1 t9	0.90	0.26	1.12	0.04
C18:1 t10	1.97	0.15	2.00	0.11
C18:1 t11	2.22	0.19	2.30	0.10
C18:1 t12	2.00	0.12	2.02	0.10
C18:1 c9	61.20	2.53	58.61	0.07
C18:1 t15	0.76	0.03	0.73	0.06
C18:1 c11	5.07	0.16	4.90	0.02
C18:1 c12	0.37	0.03	0.36	0.04
C18:1 c13	0.83	0.12	0.97	0.01
C18:2 t9t12	0.63	0.02	0.61	0.01
C18:1 t16	0.65	0.19	0.79	0.05
C18:2 c9t12	4.18	0.18	3.94	0.06
C18:2 c9c12	59.97	2.95	56.99	0.04
C18:3 n6	0.59	0.09	0.55	0.05
C18:3 t	0.00	<0.01	0.00	<0.01
C18:3 n3	0.58	0.09	0.53	0.04
C18:2 c9t11	9.01	0.63	8.25	0.13
C20:1 c9	7.93	1.16	6.85	0.07
C20:1 c11	4.39	0.53	3.70	0.16
C22	2.49	0.33	2.13	0.04
C18:3 c9t11c13	598.48	23.41	569.81	5.25
Other CLNA	178.45	29.28	160.32	11.16

μg/mg	1000.17	62.50	942.94	5.27

Fatty acid	NEFA			
	KOH (*n* = 3)		MetNa (*n* = 3)	
	Mean	SD	Mean	SD
C16	5.42	0.19	5.58	0.04
C16:1 c9	1.13	0.08	1.21	<0.01
C17	0.22	0.01	0.22	0.01
C18	1.61	0.19	1.76	0.05
C18:1 c9	6.58	0.62	7.08	0.13
C18:1 c11	0.46	0.04	0.47	0.03
C18:2 c9t12	0.19	<0.01	0.17	0.01
C18:2 c9c12	7.35	0.69	7.94	0.10
C18:3 n3	0.59	0.05	0.64	<0.01
C18:2 c9t11	0.73	0.04	0.78	0.01
C20:1 c9	0.25	0.03	0.30	0.02
C22	0.03	<0.01	0.03	<0.01
C18:3 c9t11c13	20.94	2.35	22.74	0.56
Other CLNA	12.70	1.92	14.39	0.22

μg/mg	58.20	6.21	63.30	1.12

c/t: cis/trans double bond; n6: ω6 fatty acid; n3: ω3 fatty acid.

## Appendix A Supplementary data

The following KML files contain the Google maps of the most important areas described in this article:
